# Adenovirus type 5 exerts genome-wide control over cellular programs governing proliferation, quiescence, and survival

**DOI:** 10.1186/gb-2007-8-4-r58

**Published:** 2007-04-12

**Authors:** Daniel L Miller, Chad L Myers, Brenden Rickards, Hilary A Coller, S Jane Flint

**Affiliations:** 1 Department of Molecular Biology, Princeton University, Princeton, NJ 08544, USA; 2 Laboratory of Genetics, University of Wisconsin, 425-G Henry Mall, Madison, Wisconsin 53706, USA; 3 Lewis-Sigler Institute for Integrative Genomics, Carl Icahn Laboratory, Princeton University, Princeton, NJ 08544, USA; 4 Department of Computer Science, Princeton University, Princeton, New Jersey 08544, USA

## Abstract

**Background:**

Human adenoviruses, such as serotype 5 (Ad5), encode several proteins that can perturb cellular mechanisms that regulate cell cycle progression and apoptosis, as well as those that mediate mRNA production and translation. However, a global view of the effects of Ad5 infection on such programs in normal human cells is not available, despite widespread efforts to develop adenoviruses for therapeutic applications.

**Results:**

We used two-color hybridization and oligonucleotide microarrays to monitor changes in cellular RNA concentrations as a function of time after Ad5 infection of quiescent, normal human fibroblasts. We observed that the expression of some 2,000 genes, about 10% of those examined, increased or decreased by a factor of two or greater following Ad5 infection, but were not altered in mock-infected cells. Consensus k-means clustering established that the temporal patterns of these changes were unexpectedly complex. Gene Ontology terms associated with cell proliferation were significantly over-represented in several clusters. The results of comparative analyses demonstrate that Ad5 infection induces reversal of the quiescence program and recapitulation of the core serum response, and that only a small subset of the observed changes in cellular gene expression can be ascribed to well characterized functions of the viral E1A and E1B proteins.

**Conclusion:**

These findings establish that the impact of adenovirus infection on host cell programs is far greater than appreciated hitherto. Furthermore, they provide a new framework for investigating the molecular functions of viral early proteins and information relevant to the design of conditionally replicating adenoviral vectors.

## Abstract 2

The effects of the adenovirus Ad5 on basic host cell programs, such as cell-cycle regulation, were studied in a microarray analysis of human fibroblasts. About 2,000 genes were up- or down-regulated after Ad5 infection and Ad5 infection was shown to induce reversal of the quiescence program and recapitulation of the core serum response.

## Background

The *Adenoviridae *are nonenveloped viruses of mammals and birds that are characterized by linear, double-stranded DNA genomes of 34 to 43 kilobases (kb) and strikingly icosahedral capsids that carry projecting fibers at each of the 12 vertices. Since the first adenovirus was isolated from human adenoid tissue in 1953, some 50 human serotypes have been identified and associated with various syndromes, including upper respiratory tract infections in young children, acute respiratory disease in military recruits, epidemic keratoconjunctivitis, and gastroenteritis. However, it was the demonstration that some human adenoviruses induce tumors in laboratory animals [1] that greatly increased interest in these viruses and their interactions with host cells. Although human adenoviruses can be classified as highly oncogenic, weakly oncogenic, or non-oncogenic in rodents, all transform rodent cells in culture [2].

The viral E1A and E1B early genes are necessary and sufficient for transformation, and E1A can also transform normal cells in cooperation with other oncogenes, such as activated RAS [2]. Investigation into the mechanisms by which the E1A and E1B gene products transform cells has yielded important insights into the cellular pathways that control cell cycle progression and programmed cell death, in particular the roles of the tumor suppressor proteins Rb (retinoblastoma protein) and p53 [3-7]. In addition, studies of the viral replication cycle in permissive cells have made major contributions to elucidating fundamental cellular processes, most famously with the discovery of pre-mRNA splicing [8,9].

The infectious cycle of subgroup C human adenovirus, such as adenovirus type 5 (Ad5), in permissive cells in culture is characterized by a strict temporal program of viral gene expression that culminates in production of large quantities of viral DNA and structural proteins. Viral protein encoding genes are transcribed by the RNA polymerase II transcriptional apparatus of the host, but viral proteins or processes orchestrate the strict temporal sequence in which viral genes are expressed [2]. The first to be transcribed following entry of DNA genomes into host cell nuclei is the E1A immediate early gene. The two most abundant E1A proteins, which are produced by translation of alternatively spliced mRNAs, differ only in the presence in the larger of an internal sequence of 43 amino acids. This segment is one of four E1A protein sequences that are conserved among primate adenoviruses, and denoted conserved region (CR)1 to CR4 [10-12]. It (CR3) is essential for efficient progression beyond the immediate phase of the infectious cycle because it mediates activation of transcription from viral early promoters by the larger E1A protein [13-17]. Such stimulation appears to result from the interaction of CR3 with a subunit of the mediator [18-21], a multiprotein complex that can act as a co-activator or co-repressor of transcription by RNA polymerase II [22,23].

CR2, which is common to both E1A proteins, probably contributes to activation of transcription of the viral E2 early (E2E) gene. The E2E promoter contains two binding sites for sequence-specific transcriptional activators of the E2F family [24]. The transcriptional functions of E2Fs are regulated by binding of the Rb protein, which represses E2F-dependent transcription [3-5,25]. A conserved motif within CR2 of E1A proteins binds to the same sequence of the Rb proteins as do E2Fs, and therefore can liberate E2Fs from inhibitory interactions with Rb family members. This interaction of E1A proteins with Rb is necessary for the mitogenic activity of the viral proteins in primary cells, and for their ability to transform nonpermissive cells in conjunction with the viral E1B gene or an activated RAS oncogene [26-31].

Transcription of viral early genes leads to synthesis of some 17 early proteins, many of which perturb host cell (or host) physiology. For example, several E3 proteins block host immune responses [32,33]. The two E1B proteins can protect infected cells against apoptosis. The E1B 19 kDa protein is a homolog of the cellular antiapoptotic protein Bcl, which inhibits the proapoptotic Bax [3,4,34]. In contrast, the E1B 55 kDa protein counters the consequences of activation of the tumor suppressor p53. Binding of this E1B protein to p53 converts the cellular protein from an activator to a repressor of transcription [35,36] and, in conjunction with the viral E4 orf6 protein, the E1B protein induces accelerated degradation of p53 [37-39]. Once the viral E2 replication proteins have attained sufficient concentrations, viral DNA synthesis commences. This event marks the transition to the late phase of infection and is necessary for activation of the late transcriptional program. Viral DNA synthesis dependent titration of a cellular repressor permits transcription from the promoter of the late IVa_2 _gene [40,41], which encodes a sequence-specific DNA-binding protein that has been implicated in stimulation of transcription from the major late (ML) promoter [42,43]. Transcription from the ML promoter, in conjunction with alternative processing of ML pre-mRNAs, leads to production of some 15 mRNAs, most of which encode viral structural proteins [2].

As noted above, investigation into interactions among adenoviral and cellular components has greatly improved our understanding of several fundamental processes and the impact of viral gene products on multiple cellular pathways. It has also set the stage for current efforts to develop adenovirus vectors for therapeutic applications. Much effort has been devoted to the design of vectors for delivery of endogenous genes. Within this context a major goal is to minimize host immune responses to the vector, for example by preventing expression of viral genes and viral replication [44-47]. In contrast, the development of conditionally replicating adenoviruses for selective killing of tumor cells depends on efficient replication in transformed but not in normal cells [48-50]. Nevertheless, replication of the virus in normal human cells has received little attention, despite hints of significant differences when Ad5 infects normal as compared to transformed cells. For example, the 243R protein is dispensable for Ad5 replication in transformed HeLa cells, but it is required for maximal replication in normal lung fibroblasts [30]. Similarly, the E1B 55 kDa protein is necessary for efficient viral DNA synthesis in Ad5 infected primary human fibroblasts but not in established lines of human cells [51]. Although informative, such studies of individual viral gene products cannot determine the degree to which interactions of Ad5 with host cell systems differ in established and normal cells. As a first step to address this important issue, we have undertaken a global analysis of the changes in cellular gene expression that accompany progression through the viral infectious cycle in normal human fibroblasts.

## Results and discussion

### Kinetics of the Ad5 infectious cycle

To provide a temporal framework within which to interpret changes in cellular gene expression induced by Ad5 infection of normal human fibroblasts, we first examined the accumulation of viral DNA, as well as of early and late viral proteins, as a function of time of infection. The results of preliminary experiments were used to design a time series that covered the entire infectious cycle, while focusing on the period (18 to 42 hours after infection) in which synthesis of viral macromolecules and changes in viral gene expression were maximal.

**Figure 1:**
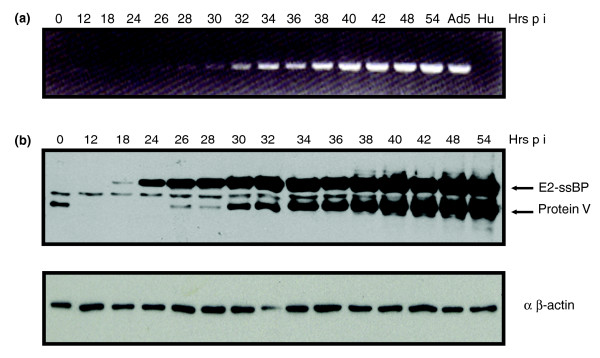
Kinetics of the Ad5 infectious cycle in quiescent HFFs. Quiescent human foreskin fibroblasts (HFFs) were infected with 30 plaque forming units/cell adenovirus type 5 (Ad5) and total DNA or protein extracts prepared after various periods of infection, as described in Materials and methods. **(a) **Concentrations of viral DNA were determined by limiting polymerase chain reaction amplification of a sequence with the E1A gene. Purified Ad5 (Ad5) and human (Hu) DNAs were amplified as positive and negative controls, respectively. **(b) **The viral early E2 and late V proteins, and the cellular β-actin protein were examined by immunoblotting.

Infected cells were harvested in parallel with those from which cellular RNA was isolated, and total DNA or protein extracts prepared from them as described in Materials and methods (below). The results of immunoblotting indicated that the viral early E2 single-stranded DNA-binding protein was present at a low concentration at 18 hours after infection, and at a substantially higher concentration by 24 hours (Figure 1a). In contrast, the late structural protein, protein V, was not clearly detected until 30 hours after infection, whereas the first increase in the intracellular concentration of viral DNA was observed between 26 and 28 hours after infection (Figure 1a,b). These data establish that in Ad5 infected, contact inhibited human foreskin fibroblasts (HFFs), the early and late phases of infection begin at around 18 hours and between 26 and 28 hours after infection, respectively. A similar time course of synthesis of other viral early and late proteins was observed when HFFs infected under the same conditions were examined by immunofluorescence (data not shown). These experiments also indicated that the viral immediate early E1A proteins, which are required for efficient transcription from all early promoters, such as that of the E2 transcription unit (see Background, above), were first made between 12 and 16 hours after infection. The onset of the synthesis of viral macromolecules is considerably delayed under these conditions as compared with the infectious cycle in established lines of human cells, such as HeLa cells, but it is very similar to that observed previously in subconfluent, proliferating HFFs [51].

### Overview of alterations in cellular gene expression induced by Ad5 infection

We conducted two-color hybridizations using Agilent 44k Whole Genome microarrays to examine time-dependent changes in the concentrations of cellular RNA species in HFFs after Ad5 or mock infection. Infected cells were harvested after various periods of infection, as described above, whereas duplicate samples of mock infected cells were collected at 24 and 48 hours. Zero time point samples for each time course (two zeros for the mock and three for the Ad5 infection) were collected immediately after the 1 hour adsorption period. Labeled cRNAs prepared from Ad5 or mock infected samples (red channel) were hybridized competitively with approximately equal concentrations of a common reference cRNA (green channel). The reference cRNA was made from a mixture of RNAs originating from a diverse set of human cells and cell lines. These differed in terms of history (primary and transformed) and proliferation state (overgrown, cycling, and quiescent), and were chosen in order to maximize the diversity of detectable cellular transcripts, by minimizing the number of probes on the arrays with below-background signals in the reference channel.

For each hybridization, variations in the input of labeled cRNA were corrected by a standard computational dye normalization (see Materials and methods, below). To facilitate comparison of the response profiles of individual probes with each other and with the temporal origin of the experiment, we zero transformed the data by probe; the log_2 _expression values of each probe in the mock and Ad5 time courses were linearly transformed by subtracting the mean values of the corresponding zero samples. Finally, to isolate a core set of probes that exhibited significant changes in expression specifically in response to Ad5 infection, we applied the following intensity filters; probes were required to exhibit a log_2 _expression value ≥ 1 (equivalent to 2-fold change) at at least three time points in the Ad5-infected series, and a log_2 _expression value ≤ 0.4 (equivalent to 1.3-fold change) in no more than two arrays in the mock infected series.

Application of these criteria identified 2,104 genes (unique Unigene clusters), of the 20,590 on the arrays, whose expression exhibited a sustained change of twofold or greater following Ad5 infection, but were not significantly altered in mock infected cells. (For a complete list of genes that pass these filters, see Additional data file 1. The complete unfiltered dataset can be accessed at the Princeton University Microarray database [PUMA] [52].)

As a comparison with our simple filter for differential expression, we applied significance analysis of microarrays (SAM) [53]. Specifically, we looked for genes that were significantly differentially regulated at two different times after infection, as compared with the triplicate zero time point measurements. To overcome the absence of replicate measurements of Ad5 infected cells, we treated the three measurements surrounding both 26 and 40 hours as triplicate measurements. Two groups of measurements, the first taken at 24, 26, and 28 hours after infection and the second group taken at 38, 40, and 42 hours, were tested independently for differential expression compared with the triplicate zero time point. To make the SAM results comparable with the results of our twofold filter discussed above, we excluded all genes that exhibited any change in response to mock infection. We then performed independent two-class unpaired analysis for the two sets of pseudo-replicates centered at 26 and 40 hours, and combined the results of these two tests. Using a false discovery rate of no more than 0.1%, we identified 5,262 genes that are differentially expressed at 26 or 40 hours after infection. This number far exceeds the 2,104 that pass the fold change filters. In addition, 96% of the genes that pass the fold change filter above were also found by the SAM analysis to exhibit statistically significant differential expression (*P *< 10^-10^). Thus, our fold change criteria defined a subset of statistically significantly differentially expressed genes that exhibit the strongest changes in mRNA levels in response to Ad5 infection.

**Figure 2:**
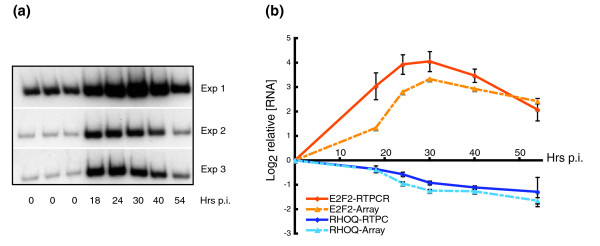
Changes in RNA concentrations in Ad5-infected HFFs determined by RT-PCR. **(a) **Autoradiograms of products of reverse transcription (RT)-polymerase chain reaction (PCR) amplification of CDC6 RNA isolated from human foreskin fibroblasts (HFFs) infected for the periods indicated. The RNA samples used in experiment 1 were those also used for amplification and hybridization to microarrays, whereas experiments 2 and 3 total RNAs were from two other independent infections of quiescent HFFs. **(b) **The RT-PCR signals for E2F2 and RHOQ RNAs from the three independent infections were quantified, as described in Materials and methods, zero transformed against the mean of the three zero time point samples included in each experiment, and converted to log_2 _values for comparison to the changes in concentration of these RNAs determined by hybridization to microarrays. Ad5, adenovirus type 5.

The concentrations of a subset of the cellular RNAs that exhibited changes in response to Ad5 infections satisfying our fold change filter were determined using an alternative method, namely reverse transcription (RT)-polymerase chain reaction (PCR). In parallel, we examined the same RNAs in samples isolated at various times after two additional and independent infections of HFFs. Representative results of the RT-PCR experiments are shown for CDC6 RNA in Figure 2a. Although the absolute quantities of CDC6 RNA present after increasing periods of infection varied among infections, the temporal patterns of changes in concentration were the same in all cases. The gene expression patterns for 16 different genes were determined by RT-PCR. The data were zero transformed and converted to log_2 _values for comparison with the microarray data (Figure 2b and Additional data file 2). The two methods of analysis yielded closely similar patterns of temporal changes in expression for 14 of the 16 genes examined (87.5%), as illustrated in Figure 2b for two RNAs that differed in direction as well as magnitude of Ad5-induced alterations. These results establish both the reproducibility of the changes in cellular gene expression induced by Ad5 infection of quiescent HFFs and the reliability of the alterations detected by hybridization to microarrays.

**Figure 3:**
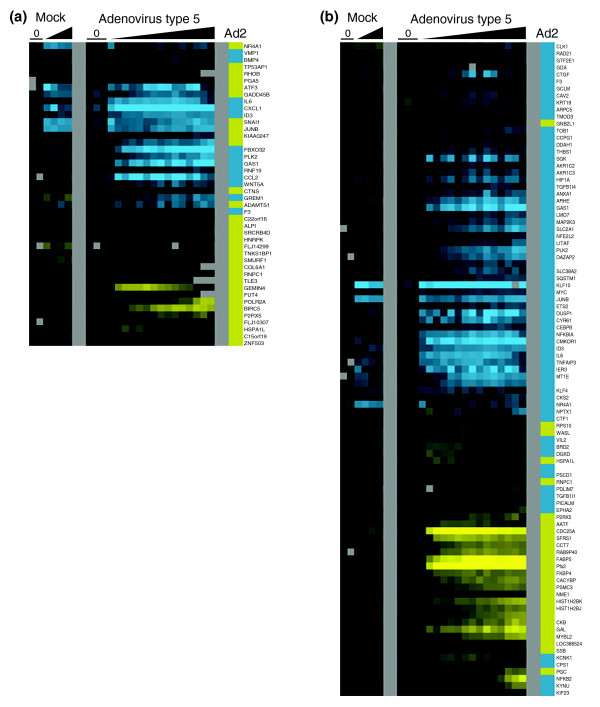
Comparison of adenovirus-induced changes in gene expression in HeLa cells and HFFs. The genes reported to exhibit changes in expression at **(a) **6 hours or **(b) **10 and 21 hours after infection of HeLa cells by adenovirus type 2 (Ad2) [54,55] were isolated from our dataset and clustered on the basis of their responses to infection of human foreskin fibroblasts (HFFs). The changes observed in HeLa cells are summarized in the columns labeled Ad2, in which yellow and blue represent increased and decreased expression respectively. In panel b, the HeLa response is based on the average of the two time points. Ramps above panels indicate increases in time after infection.

In addition to confirming our results by statistical and quantitative means, we wished to compare them with those of published reports of changes in cellular gene expression induced by adenovirus infection. During the early phase of Ad5 infection of transformed HeLa cells, expression of 76 cellular genes of the 12,309 examined was observed to be increased or decreased by a factor of 1.5-fold or greater, whereas expression of 112 genes was specifically altered during the late phase of infection [54,55]. The majority of these RNAs exhibited similar alterations in concentration following Ad5 infection of HFFs (Figure 3). However, expression was modulated for a significantly larger proportion of the cellular genes examined in Ad5-infected HFFs than in HeLa cells (10.5% versus about 1.5%).

Differences in the methods used to collect and analyze hybridization data are likely to contribute to these seemingly disparate responses to infection, as are the genetic histories of the infected cells. Highly transformed and genetically abnormal cells, such as HeLa cells, are likely to be less sensitive to stresses such as viral infection, and may have lost cellular systems that are targeted by adenovirus in normal diploid fibroblasts. For example, HeLa cells contain integrated copies of the human papillomavirus type 18 oncogenes encoding the E6 and E7 proteins [56], which, like adenoviral E1B and E1A proteins (see Background, above), inactivate the cellular tumor suppressors p53 and Rb, respectively [57-59]. However, as discussed below, an important determinant of the extent to which cellular gene expression is reprogrammed in Ad5 infected cells appears to be whether cells are proliferating (subconfluent HeLa cells) or quiescent (contact-inhibited HFFs) at the time of infection. In addition to a larger number of responsive genes, our analysis of cellular RNA concentrations at many time points has identified multiple temporal responses to Ad5 infection.

### Clustering of co-regulated genes

In order to identify groups of genes that exhibited significant co-regulation, we chose to apply a k-means clustering algorithm to the filtered dataset. The clustering process begins by randomly assigning all genes to k total clusters and computes a centroid vector for each cluster. The algorithm then iteratively reassigns genes to clusters based on the closest match (highest Pearson correlation) of individual expression vectors to the cluster centroids, until no changes allow better matches of gene to cluster means. To ensure that the final clustering result was not sensitive to the initial, random assignment of centroids, we report the consensus of 5,000 such runs of k-means clustering. We determined an appropriate number of clusters, k, by using figure of merit (FOM) analysis, which measures the predictive power of a clustering result by leaving one condition out of the clustering process and measuring how predictive the cluster centroids are of the held-out condition [60]. Details of the consensus clustering are discussed in Materials and methods (below).

**Figure 4:**
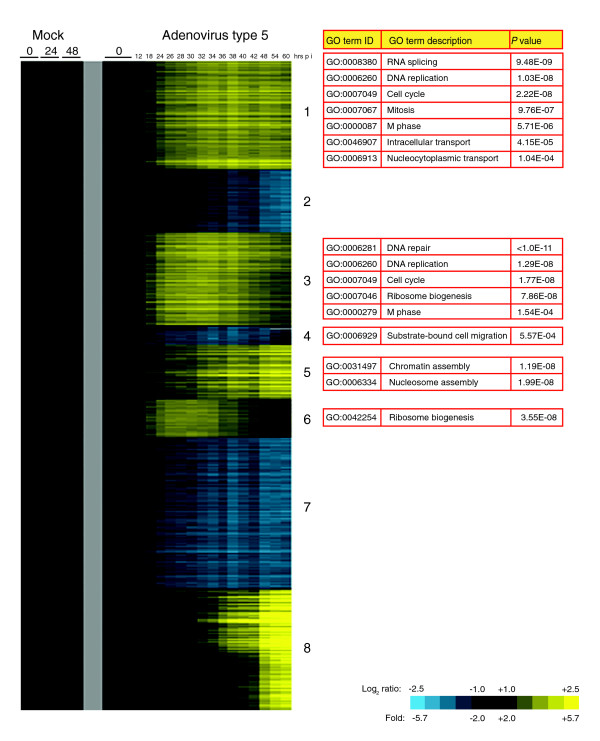
Kinetic patterns of expression of Ad5-responsive genes and associated cellular functions. The log_2 _expression values of the 2,106 genes that passed the filters described in the text clustered into eight groups are shown at the left, and over-represented Gene Ontology (GO) terms in each cluster at the right. Also shown is a color-bar relating both log_2 _ratios and fold changes (relative to the average zero values) to color intensity. Ad5, adenovirus type 5.

In this way, eight groups of genes were clearly distinguished on the basis of the temporal patterns of alterations in the corresponding RNA concentrations induced by Ad5 infection (Figure 4). Although approximately equal numbers of cellular RNA species represented in the total dataset increased or decreased in concentration during the viral infectious cycle (data not shown), nearly two-thirds of the probe responses in the filtered list exhibited an increase in RNA concentration. Those exhibiting a reduction in concentration fell into three clusters, distinguished primarily by the time after infection at which a significant change in RNA concentration was first detected: early after infection, 24 hours (cluster 7), or late in infection, between 32 and 36 hours (cluster 2). A third cluster of downregulated genes (cluster 4) consisted of a small number of genes encoding RNAs that decreased in concentration early in infection, reached their lowest levels between 34 and 38 hours after infection, but returned to baseline concentrations by the end of infection (Figure 4). In contrast, the kinetic patterns of increases in cellular RNA concentrations were considerably more variable, in terms of both the time after infection at which an increase was first detected and the duration of the change (Figure 4). For example, increased accumulation of a substantial number of cellular RNAs was evident by 18 or 24 hours after infection, but in some cases RNA concentrations subsequently decreased (clusters 3 and 6), whereas in others the initial alteration was sustained (cluster 1) or amplified (cluster 5) later in the infectious cycle. The number of RNA species observed to increase in concentration during the late phase of infection was relatively small (about 500; Figure 4). Unexpectedly, however, the maximal increases in accumulation of these RNAs were observed very late in the infectious cycle, from 48 hours after infection (clusters 8), when infected cells are largely devoted to assembly of virus particles.

### Analysis of cellular functions targeted by Ad5 infection

In an attempt to identify cellular functions that are predominantly affected by Ad5 infection, we searched the filtered list of genes significantly altered in expression for statistical over-representation of functional classes. We used a local implementation of GoTermFinder (see Materials and methods, below), which maps each gene in a query list to a node in the 'biologic process' ontology of the Gene Ontology Consortium [61], and computes a probability for the preponderance of each function in the query list. To avoid a possible function bias in the population of genes present on the arrays, the *P *value for over-representation was computed using all genes on the array as the background population. In addition, the *P *value was Bonferroni-corrected for multiple hypothesis testing. In this way, several important cellular functions were found to be modulated specifically in response to Ad5 infection (Table 1). We wished to determine whether the specific targeting of cellular functions correlated with temporal patterns of changes in gene expression, and therefore searched the kinetic clusters generated by consensus k-means clustering (Figure 4) for over-represented function terms.

Several instances of Ad5-induced co-regulation of genes associated with common cellular functions were identified (Figure 4). Cellular RNAs that increased in concentration slowly and steadily throughout most of the observed infectious cycle, which are grouped in cluster 5, exhibited a highly significant enrichment in RNAs specifying proteins that participate in the establishment and maintenance of chromatin structure (*P *= 10^-8^). These RNAs encode chromatin modifying proteins, such as the histone methyl transferase DotIL and subunits of the NuAY histone acetyl transferase, and numerous core histones, including the S-phase specific histone H2BFS. Cluster 1 contains RNAs that increased in abundance early in response to Ad5 infection and remained elevated thereafter. Genes encoding RNA splicing components are significantly enriched in cluster 1 (*P *= 10^-8^), and these RNAs encode several snRNP core Sm and Sm-like protein proteins, SF3A subunits, and proteins critical for enhancer mediated splicing. RNAs encoding proteins that mediate nucleocytoplasmic transport of both RNA and protein molecules, such as importins and nucleoporins, were also over-represented in cluster 1 (*P *= 10^-4^).

As discussed above, cellular RNAs that exhibited strong, Ad5-induced increases in concentration early in infection (18 to 24 hours) fell into three main kinetic clusters that differ in terms of whether initial increases in RNA concentration were maintained for the duration of the viral life cycle (cluster 1) or steadily declined from 34 hours after infection (clusters 3 and 6). Despite differences in temporal patterns of expression, these clusters exhibited common enrichment in genes ascribed important functions related to cellular proliferation (Figure 4). Both clusters 1 and 3, which are distinguished by whether initial increases in RNA concentration were sustained throughout the infection, were strongly enriched in genes specifying proteins that function in progression through the cell cycle (*P *= 10^-8 ^in both). These include checkpoint proteins, DNA replication licensing proteins, and cell cycle promoting transcription factors. Enrichment for increased expression of such cell cycle genes is consistent with the well established mitogenic activity of viral E1A proteins (see below). Furthermore, genes encoding proteins that mediate or regulate DNA replication were also highly enriched in clusters 1 and 3 (*P *= 10^-8 ^in both), as were genes associated with M phase (*P *= 10^-5 ^and *P *= 10^-4^, respectively). These include genes encoding subunits of DNA polymerases, and Mcm complex components, as well as subunits of the anaphase promoting complex, mitotic checkpoint proteins, and proteins that regulate spindle formation, chromosome condensation and chromosome segregation. Interestingly, cluster 3 is also highly enriched for transcripts of DNA repair genes (*P *< 10^-11^). In particular, the Fanconi anemia group pathway of DNA repair is heavily targeted by Ad5 infection, as are the central pathways required for the detection and signaling of DNA damage, represented by the catalytic subunit of DNA-dependent protein kinase and the UV damage sensor Rad18. Finally, cluster 6 differs from cluster 3 in that the peak expression levels reached early after infection, subsequently decreased far more dramatically than those in cluster 3, and returned to near baseline levels by 60 hours. Nevertheless, both cluster 3 and 6 are highly enriched for genes involved in another cellular function important for growth and proliferation, namely ribosome biogenesis (*P *= 10^-7 ^and *P *= 10^-8^, respectively). Nucleolar proteins feature prominently in these two clusters, including many that participate in pre-rRNA biosynthesis and maturation, as well as ribosome particle assembly.

Assuming that changes in cellular gene expression induced by Ad5 infection result in corresponding increases or decreases in protein production, and hence activity, we can conclude that the diversity of cellular functions modulated during the adenoviral life cycle is far greater than was previously appreciated. Perhaps even more striking is the substantial enrichment in the 2,000 or so RNAs that changed most strongly in concentration in Ad5-infected HFFs, with those that encode proteins that mediate and regulate cell cycle progression and cell proliferation (Figure 4). Because the HFFs were quiescent at the time of infection, this finding prompted us to undertake a comparison of alterations in gene expression induced by infection and by entry into, and exit from, the quiescent state.

#### Ad5 infection induces reversal of the quiescence program and recapitulation of the core serum response

**Figure 5:**
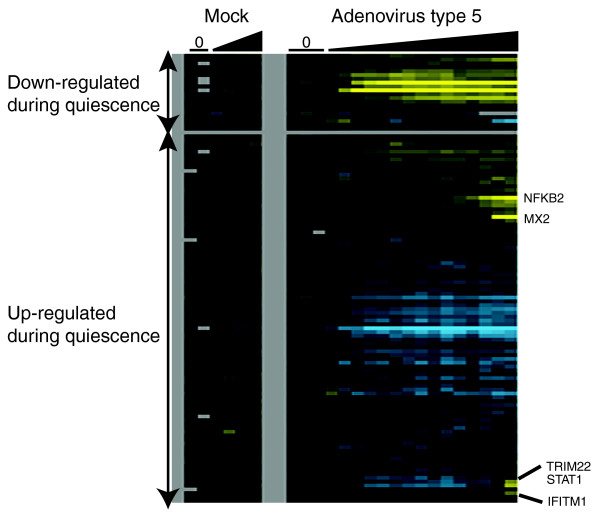
Ad5 infection induces reversal of the quiescence program. The expression responses of the quiescence program genes to adenovirus type 5 (Ad5) infection were isolated, and divided into two groups on the basis of expression changes during quiescence. The members of each group (downregulated during quiescence and upregulated during quiescence) were then hierarchically clustered and the two groups then rejoined for visualization. The genes named at the right are discussed in the text. Ramps above panels indicate increases in time after infection.

Upon reaching confluence in tissue culture, primary fibroblasts undergo a highly regulated transition into a reversible growth arrest termed quiescence. Recently, the core alterations in gene expression that accompany this process in diploid human fibroblasts were defined [62]. To determine whether the genes associated with the induction of the quiescence program exhibited any systematic changes in expression after Ad5 infection, the expression changes of the genes that are specifically upregulated or downregulated as primary lung fibroblasts become quiescent were linked to the expression changes in response to Ad5 infection of quiescent HFFs. The data were then organized by regulation of gene expression during quiescence (Figure 5).

Strikingly, genes encoding RNAs that decreased in concentration during quiescence were preferentially upregulated during infection, whereas the transcripts of genes activated during quiescence exhibited nearly systematic Ad5-induced decreases in abundance. To test whether this opposing pattern was statistically significant, we clustered the infection responses into two groups, namely those exhibiting an upward and those exhibiting a downward trend (data not shown), and used the hypergeometric probability distribution to compute *P *values for the nonrandom representation of quiescence RNAs in each cluster. Downregulated quiescence genes were significantly enriched in the cluster of Ad5 upregulated responses, and upregulated quiescence genes in the cluster of Ad5 downregulated RNAs (*P *7 × 10^-4 ^and 9 × 10^-5^, respectively). Furthermore, the set of genes that did not conform to the reverse pattern (< 30% of the quiescence program genes) generally exhibited weak responses to Ad5 infection and were less than half as likely to pass cutoff filters compared with the set of genes that did conform to the pattern of quiescence reversal. In addition, the several genes that were upregulated both during quiescence and late in Ad5 infection encode proteins (myxovirus resistance protein 2, NF-kappa-B2 [p49/p100], Trim22, and Stat1) that are components of the host antiviral defense mediated by interferon [63,64].

In all, our findings show a robust reversal of the expression profile recently identified as the core signature of quiescence. The late changes in RNA concentration, and the cellular function of the corresponding genes that do not conform to this reversal, suggest that their increased expression is part of a general response to infection, superimposed on the Ad5-specific reversal of the quiescence program.

Prolonged serum withdrawal represents one way in which quiescence can be induced in primary cells. Upon re-addition of serum, the quiescent state is reversed, and cells re-enter the cell cycle, accompanied by profound changes in their gene expression profile. In fibroblasts, these changes clearly reflect their role in wound healing [65]. The apparent reversal of the quiescence gene expression program induced by Ad5 infection suggested that infection of quiescent fibroblasts by Ad5 may represent an event akin to serum stimulation. To test this hypothesis, we isolated the Ad5 induced changes in expression of the core serum response (CSR) signature genes identified by Chang and coworkers [66,67]. In remarkable studies, those researchers showed that primary human fibroblasts from different parts of the body exhibit distinct location specific expression profiles [66], yet they share a common transcriptional response to serum [67]. The latter is termed the CSR, and is defined as the set of 512 genes that, in all 50 fibroblast lines from 10 different anatomical sites, exhibit differential expression when cultured in the presence or absence of serum. Importantly, genes that had been found to show a periodic pattern of altered expression during the cell cycle [68] were excluded from this list. Thus, the CSR represents a stereotyped, serum dependent gene expression signature, which is independent of cell cycle associated responses to serum.

**Figure 6:**
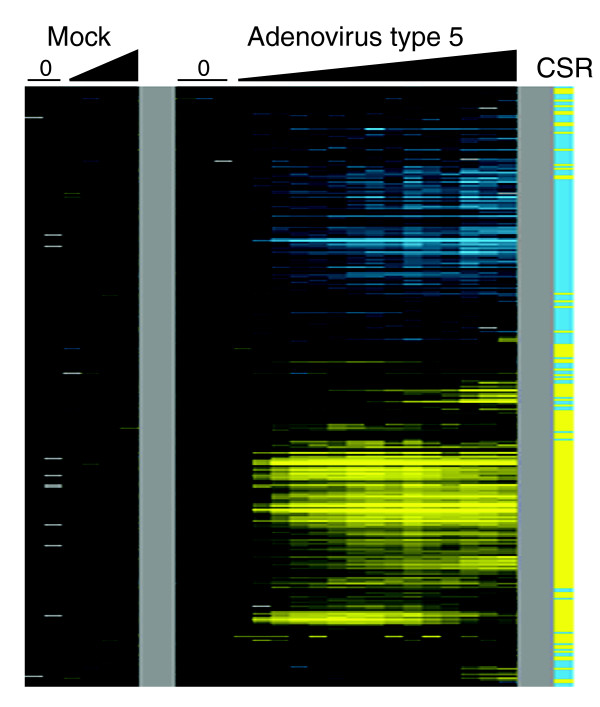
Induction of the core serum response by Ad5 infection. The responses of the core serum response (CSR) genes to adenovirus type 5 (Ad5) infection were isolated and clustered on the basis of their response to Ad5 infection. The serum dependent expression responses are summarized in yellow or blue (activated or repressed, respectively) in the rightmost column labeled CSR. Ramps above panels indicate increases in time after infection.

We hierarchically clustered the genes that comprise the CSR on the basis of their alterations in expression during Ad5 infection of HFFs (Figure 6). This approach established that increases in expression in response to infection are highly correlated with elevated gene expression in the presence of serum. Similarly, Ad5 induced decreases were correlated with lower expression in response to serum (Figure 6). Indeed, the concordance between the two datasets for this group of genes is remarkable (*P *< 10^-34 ^and *P *< 10^-14 ^for RNAs that decreased and increased in concentration during Ad5 infection, respectively). These findings strongly suggest that Ad5 infection not only elicits a reversal in the gene expression program that is characteristic of quiescent human fibroblasts, but also induces a nearly perfect recapitulation of the highly specific core gene expression profile exhibited by cells that proliferate in the presence of serum growth factors. Underscoring its close association with the wound healing program, the CSR is significantly enriched for genes encoding proteins that participate in blood coagulation, complement activation, and angiogenesis, and contains genes associated with cell motility, extracellular matrix remodeling, and the myofibroblast phenotype [67]. Thus, in addition to the induction of proliferation (see above), it appears that Ad5 infection induces a cellular state closely associated with a specialized, serum dependent fibroblast function.

In addition to providing a molecular fingerprint of fibroblasts that grow in the presence of serum, the transcriptional CSR phenotype has been shown to be a robust predictor of the clinical outcome of several human carcinomas [67]. The metastasis and death associated with these human tumors correlated strongly with expression of the CSR phenotype. Thus, even though adenovirus infection is not tumorigenic in humans, it appears that infection of human cells induces a transcriptional state associated with aggressive tumor progression and poor clinical outcome.

### Mechanisms by which Ad5 gene products regulate cellular gene expression

#### The E1A proteins

Internalization of adenovirus particles by receptor mediated endocytosis is initiated by binding of the capsid penton base to cell surface α_*'i *_integrin molecules [2]. This interaction also results in very rapid (within 10 min), transient activation of phosphoinositide-3-OH kinase [69]. Although activation of signaling via this enzyme has the potential to alter gene expression [70-73], we detected no changes in cellular RNA concentrations during the first hours of the infectious cycle (Figure 4). Rather, the earliest alteration detected took place at 18 hours after infection (Figure 4), coincident with the onset of viral early gene expression (Figure 1). These changes in cellular gene expression are almost certainly the result of changes in rates of transcription; post-transcriptional mechanisms that govern RNA production in Ad5-infected cells operate only late in infection (see Background, above). Furthermore, the adenoviral immediate early E1A proteins, which are necessary for efficient progression beyond the initial phase of the infectious cycle, can regulate transcription by multiple mechanisms. In view of the findings reported above, the effects of CR2 of E1A proteins on cellular E2F proteins were of particular interest.

The E2F transcriptional regulators were first identified, and named, by virtue of their ability to bind specifically to two sites in the type C adenoviral E2 early promoter [24]. This family is now known to comprise at least eight members, which differ in their association with Rb family members, effects on transcription, and mechanisms of binding to DNA [74-78]. For example, E2F-1, E2F-2, and E2F-3 bind directly to the Rb protein and are strong activators of transcription. They are also necessary for activation of E2F-responsive genes and entry into S phase [79]. E2F-4 and E2F-5 can also associate with other members of the Rb family and stimulate transcription less strongly, whereas E2F-6, E2F-7, and E2F-8 appear to repress transcription. Association of E2F proteins with Rb, which inhibits expression of E2F responsive genes, is normally regulated during the cell cycle by phosphorylation of Rb [80]. However, CR2 dependent binding of E1A to Rb protein induces release of E2F, and hence activation of transcription of E2F responsive genes and induction of progression into the S phase of the cell cycle [3,81,82]. These effects of CR2, results discussed in the previous section, and the observation that RNAs encoding E2F-1 and E2F-2 increased significantly in concentration from 24 hours after Ad5 infection, whereas E2F-4, E2F-5 and E2F-7 RNAs did not, suggested that E2F-responsive genes were likely to be highly targeted in Ad5 infected HFFs.

A common method used to associate particular transcriptional regulators with induction of specific patterns of gene expression is to search the regulatory sequences of co-regulated genes for statistically significant over-representation of binding sites for such proteins. An initial, manual search of the entire set of genes from positions -1,000 to +500 for the two most common variants of an 8 base pair (bp) E2F consensus binding site [83,84] identified a large number of potential E2F-responsive genes (some 500). Nearly 80 of these were genes were in clusters 1, 3, and 4, which contain genes specifying RNAs that increased in concentration in response to Ad5 (data not shown). However, in the case of E2F, methods based on identification of binding site sequences are problematic; the currently defined consensus binding sites for members of this family includes a significant degree of degeneracy [83,84]. Furthermore, it has recently been reported that many sequences to which E2F binds *in vivo *do not match such consensus sequences [85]. We therefore examined the effects of Ad5 infection on the expression of genes to which E2F family proteins are known to bind *in vivo*, which have been identified by immunoprecipitation of E2F containing chromatin and microarray analysis of the DNA [86].

**Figure 7:**
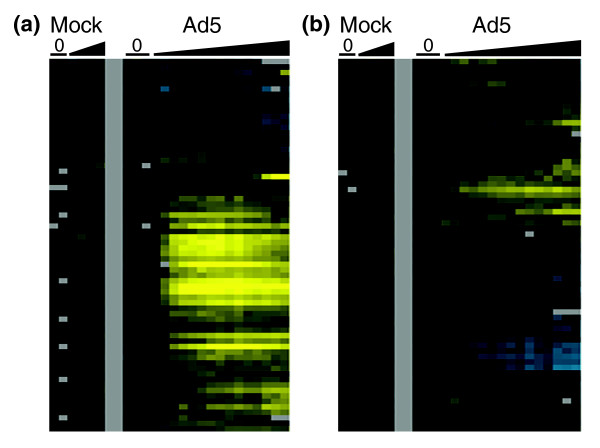
The expression of E2F target genes in Ad5 infected HFFs. **(a) **The 67 genes bound by E2F2 or E2F4 identified by Ren and coworkers [86] and **(b) **67 randomly chosen genes were isolated from the complete dataset while excluding genes with responses to mock treatment. The genes in both panels were hierarchically clustered for easier viewing. Ramps above panels indicate increases in time after infection. Ad5, adenovirus type 5; HFF, human foreskin fibroblast.

From our complete, that is, unfiltered, dataset we isolated the expression changes of the direct E2F target genes identified by Ren and coworkers [86], while excluding genes that exhibit changes in expression in response to mock infection. Comparison with an equal number of randomly selected genes from this dataset revealed strong enrichment of Ad5 responsive genes among direct E2F target genes (Figure 7). In fact, 60% of the 67 E2F target genes that showed no significant response in mock infected cells passed the stringent fold-change filtering scheme applied previously (compared with 10% of all the genes in the dataset), and all but seven are grouped in clusters 1 and 3 (Figure 4) with the genes that exhibit the earliest activation in response to Ad5 infection (data not shown).

**Figure 8:**
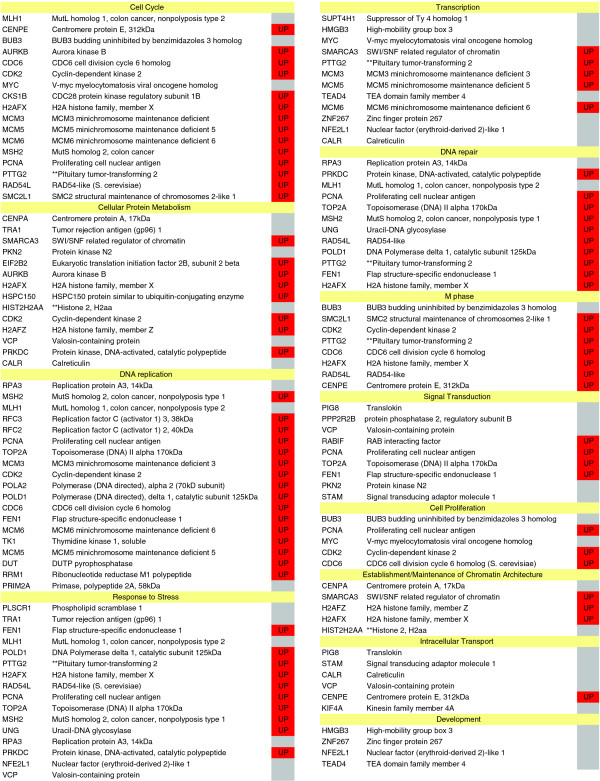
Ad5 induced changes of expression of E2F target genes organized by function. E2F target genes, identified by Ren and coworkers [86], were organized by cellular function. Genes significantly regulated by Ad5 (those that pass fold change filter applied previously) are indicated with red/UP or green/DOWN. Grey indicates genes not significantly regulated by Ad5. (Note that none of the 67 E2F target genes were significantly downregulated.)

We then tabulated the Ad5 specific expression responses of the E2F target genes according to function (Figure 7). Genes that passed the fold change filter applied previously were labeled as responsive, either up or down. Across all functions, a significantly higher proportion of E2F target genes showed significant changes in the concentrations of their RNA transcripts in Ad5 infected HFFs, relative to all genes in the dataset. Moreover, the expression of all E2F target genes increased, rather than decreased, in infected cells. It is therefore likely that during infection of nonproliferating cells, effects mediated by CR2 of the E1A proteins account for an important subset of the changes in cellular gene expression summarized in Figure 3, as predicted by current models [3,81,82]. Unexpectedly, however, we observed that the expression of some functional groups of genes bound by E2F was more heavily affected than that of others (Figure 8). Notably, E2F targets encoding proteins that mediate or regulate cell cycle progression and DNA replication and repair exhibited much greater propensity to change in response to Ad5 than did genes associated with transcription, intracellular transport, or development (Figure 8). These different responses might be the result of preferential recognition of E2F responsive promoters by different E2F family members or effects of other transcriptional regulators that also recognize such promoters. Regardless, such function specific, differential regulation of E2F target genes suggests that additional mechanisms must regulate expression of E2F responsive genes, or E2F proteins, in adenovirus infected cells.

The E2F responsive genes described above represent but a subset of those that increased or decreased in expression during the early phase of Ad5 infection. However, E1A proteins can modulate cellular transcriptional regulators by at least three additional mechanisms. Because the interaction of CR3 of the large E1A protein with the mediator complex (see Background, above) both stimulates transcription by RNA polymerase II *in vitro *[21] and is necessary for efficient transcription of viral early genes in infected cells [20], it may well contribute to activation of cellular gene expression. CR1 and CR4 of the E1A proteins associate with various histone acetylases that activate transcription [3] and the transcriptional co-repressor Ct-BP [87,88], respectively. The latter interaction inhibits E1A dependent transformation, whereas CR1 is necessary for transformation. Neither the transcriptional consequences of association of CR1 or CR4 with cellular co-activators or co-repressors, nor the contributions of these E1A sequences to the viral infectious cycle are understood. However, these interactions could well result in either increases or decreases in transcription of specific cellular genes. It will therefore be of interest to examine the effects of substitutions that block the interactions of these conserved regions of E1A proteins with cellular components on cellular gene expression.

#### The E1B 55 kDa protein

Like E1A proteins, the E1B 55 kDa protein can also modulate cellular gene expression by multiple mechanisms. In terms of molecular detail, the best understood is repression of p53-dependent transcription [35,36]. Such inhibition of the transcriptional function of this cellular tumor suppressor protein is mediated by binding of the viral protein to the activation domain of p53 [89-91]. The results of *in vitro *experiments indicate that, when associated with p53, the E1B protein actively represses transcription [89,90]. It is therefore believed that the carboxyl-terminal repression domain of the E1B 55 kDa protein is recruited to specific promoters via the DNA binding activity of p53 to repress transcription of p53 responsive genes. Consistent with this model, substitutions or insertions at specific positions within the E1B repression domain prevent inhibition of transcription in transient assays or in *in vitro *reactions [35,89]. These mutations also impair the ability of the E1B gene to cooperate with E1A in transformation of rodent cells. In addition, it has been reported that the E1B 55 kDa protein inhibits acetylation of p53 by the histone acetyltranferase PCAF [92], a modification that is important for activation of p53 [93]. The viral protein also prevents stimulation of p53 dependent transcription in transient expression assays by the cellular protein Daxx [54]. However, it is not known whether p53 dependent transcription is repressed during Ad5 infection, as these observations predict. We therefore wished to exploit the data described previously to begin to address this issue.

**Figure 9:**
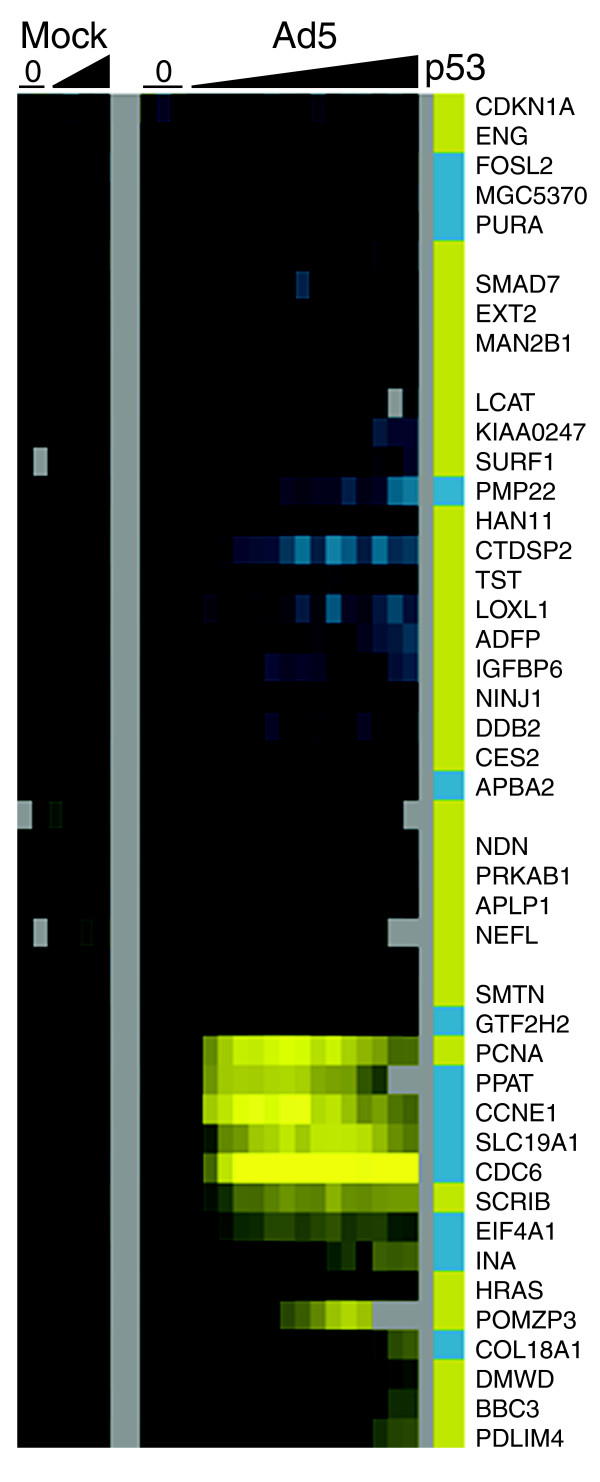
Changes in expression of direct p53 target genes induced by Ad5 infection. The 50 or so genes that are direct p53 targets in human lung fibroblasts [94] are shown clustered based on the changes in their expression observed in adenovirus type 5 (Ad5) infected human foreskin fibroblasts. The column labeled p53 summarizes the p53 induced alterations in expression of these genes, which are listed at the right. Ramps above panels indicate increases in time after infection.

Initial inspection of classical, p53 activated genes indicated that Ad5 infection induced decreases (for example, CDKN7A and all cyclin G genes) or no change (for example, BAX and MDM2) in the concentrations of corresponding RNAs. To conduct a systematic analysis, we took advantage of a careful microarray study conducted by Kannan and coworkers [94], in which they identified a set of primary p53 target genes using a temperature sensitive p53 protein synthesized in a p53 null human cancer cell line. This group identified a core set of primary p53 target genes that exhibited significant expression changes when cells were shifted to the permissive temperature, both in the presence and absence of cycloheximide. This approach, which excludes indirect p53 responses that are likely to be dependent on protein synthesis, identified approximately 50 genes as primary targets. We therefore isolated these genes in our dataset. We found that the vast majority of primary p53 target genes exhibited either no response to Ad5 infection or, most frequently, a reversal of p53 induced changes (Figure 9). Thus, most genes activated by p53 were either repressed upon Ad5 infection or unchanged in expression. On the other hand, genes normally directly repressed by p53 were de-repressed during adenovirus infection. We therefore conclude that that Ad5 orchestrates an extremely effective suppression of p53 transcriptional activity. These observations provide the first evidence suggesting that the E1B 55 kDa protein counters the transcriptional function of activated p53 in Ad5 infected cells.

## Conclusion

One of the most remarkable conclusions to emerge from the global analysis of the responses of quiescent fibroblasts to Ad5 infection presented here is that a small number of viral gene products can induce massive reprogramming of cellular gene expression. Even with application of stringent filters, some 10% of the 20,000 or so human genes examined increased or decreased in expression specifically in Ad5 infected cells. Our data also indicate that previously described properties of viral early proteins, such as binding of E1A gene products to Rb family members, are likely to account for the responses of particular sets of cellular genes. Nevertheless, in terms of explanatory power, such well characterized functions of viral proteins represent but the tip of the iceberg, for they apply to no more than 5% of the changes observed. It is probable that effects of activities of viral proteins that are not yet well understood, for example the interaction of CR1 of E1A proteins with cellular transcriptional co-activators, contribute to the reprogramming of cellular gene expression. Secondary consequences of the effects of viral early proteins are also likely to contribute via induction of transcriptional cascades.

It is also apparent that a significant fraction of the alterations in cellular gene expression represent Ad5-induced reversal of the quiescence program with concomitant induction of the core serum response, and activation of expression of many genes associated with cell proliferation. These observations provide the first experimental support for the long-held view that the mitogenic activity of adenoviral E1A proteins, which is crucial for transformation of nonpermissive cells, optimizes the environment of permissive cells for viral replication. Clearly, it will be important in future experiments to examine both cellular responses and progression through the infectious cycle in quiescent fibroblasts infected by viruses carrying mutations that impair specific functions of these viral early proteins. Such information should facilitate design of adenoviral vectors for therapeutic applications.

## Materials and methods

### Cells and virus

Human Ad5 was propagated in HeLa cells in suspension culture, as described previously [95]; purified by adsorption to an AdenoPack™ membrane (Sartorius, Goettingen, Germany), in accordance with the manufacturer's instructions; and titered by plaque assay on HeLa cell monolayers [96]. Primary HFFs were maintained in monolayer culture in Dulbecco's modified essential medium (GIBCO-BRL, Gaithersburg, MD, USA) supplemented with 10% (vol/vol) fetal bovine serum (Gemini, West Sacramento, CA, USA). Before Ad5 infection, HFFs were cultured in six-well dishes until 3 days after they had become fully confluent. They were then infected with 30 plaque forming units/cell of Ad5, a multiplicity previously determined to be sufficient for 100% infection of HFFs [51], or were mock infected. After adsorption of virus for 1 hour at 37°C, the innoculum was removed and replaced with the original, conditioned medium. Mock and Ad5 infected cells were incubated at 37°C.

### Analysis of viral DNA and protein synthesis

Infected cells were scraped from individual wells of six-well dishes after various periods of infection, washed twice in ice cold phosphate-buffered saline, and divided into two equal portions. Total DNA was purified from one portion and the quantity of viral DNA examined by using 12 cycles of PCR amplification of a segment of the viral E1A gene, as described previously [51]. The amplified DNA was analyzed by electrophoresis in a 1.4% (weight/vol) agarose gel cast and run in 0.04 M Tris-acetate (pH 7.5) containing 1 mM EDTA. A total protein lysate was prepared from the second portion of each sample of cells, as described previously [97]. The viral early E2 single-stranded DNA-binding protein and late protein V were detected by immunoblotting with monoclonal antibodies B6 [98] and F58#1 [99], respectively, as described previously [51].

### Analysis of cellular gene expression

Ad5 infected and mock infected HFF cells were harvested for RNA isolation using the RNeasy Micro™ kit (Invitrogen, Carlsbad, CA, USA), following the manufacturer's instructions. In brief, medium was completely removed from wells followed by immediate addition of a denaturing lysis buffer (RLT™, supplied by the manufacturer), homogenization by vortexing, and freezing on dry ice. All samples were thawed and processed for RNA isolation in parallel. The purification included an on-column DNAse I digestion step. The yield and quality of each RNA sample were assessed by nano-drop spectrophotometry and agarose gel electrophoresis, respectively. For each sample, 400 ng RNA was linearly amplified and labeled in the presence of Cy5-CTP, using Low RNA Input Linear Amplification reagents (Agilent Technologies, Santa Clara, CA, USA). Amplified RNA was purified on RNAeasy™ spin columns (Qiagen, Valencia, CA, USA). A mixture of total cellular RNA from five different types of human cells, including both transformed cell lines and primary cells, was labeled with Cy3-CTP and purified in the same way for use as a common reference. The amplification/labeling reactions yielded specific activities of 10 to 12 pmol Cy3/Cy5 per μg cRNA. Samples were processed and hybridized to Agilent Whole Human 44 k DNA microarrays for 17 hours at 60°C in parallel staggered batches using the Agilent hybridization kit. Slides were washed in parallel according to the manufacturer's protocol, which included a final drying rinse in acetonitrile, and scanned in batch using an Agilent two color scanner. All pre- and post-hybridization procedures with labeled cRNAs were performed in an ozone free facility.

### Data normalization and processing

Raw image data were extracted using Agilent Feature extraction software with the protocol settings recommended by the manufacturer. Raw channel intensities were adjusted for background using a spatial de-trend algorithm, dye normalization was performed using an intensity-dependent lowness normalization based on spots that passed a rank consistency filter, and final spot values were computed as the log_2 _of processed channel intensity ratios (red/green). The extracted data were loaded onto the Princeton University PUMA database [52] for storage and spot quality filtering. Spots flagged by the extraction software as feature uniformity outliers, in either channel, were culled. In addition, only spots flagged as well above background were included in the analysis; uncorrected channel intensity values passed a two sided *t*-test (*P *= 0.01) for significance, and background-corrected signals were greater than 2.6 times the standard deviation of global background. Processing of corrected and filtered log_2 _dye ratio data, including dataset manipulations, zero transformation, filtering, and visualization, were performed using the following freeware: PCL-Analysis (Gavin Scherlock, Stanford University, Palo Alto, CA, USA), TIGR MeV data analysis package, and Java-Treeview (Alok Jerome Saldanha, Stanford University, Palo Alto, CA, USA). Primary probe accessions supplied by Agilent Technologies were mapped to Unigene clusters via batch retrieval using SOURCE [100] using the Protein Information Resource batch retrieval utility [101]. To establish a dataset of unique genes, the expression values associated with multiple probes referencing the same gene were averaged.

### RT-PCR analysis

Cellular RNA (100 ng) isolated from HFF cells at 0, 18, 24, 30, 40 and 54 hours after infection (as described in a previous section) was analyzed by RT-PCR for several human genes using the RT-PCR Master Mix (2 ×) kit (USB, Cleveland, OH, USA) in the presence of 3 μCi/reaction [α^32^P]-dATP (3000 Ci/mmol; Perkin Elmer, Waltham, MA, USA). For each gene, the optimal number of cycles was experimentally determined to ensure exponential amplification. The primer pairs, ranging in size from 19 to 24 bases, were designed to amplify GMNN (positions +68 to +347 of the coding sequence), CCNE1 (+79 to +314), E2F2 (+1,133 to +1,344), MSH2 (+807 to +1,053), SFRP1 (+1,366 to +1,609), FAM45A (+606 to +759), NOLC1 (+639 to +886), LENG9 (+448 to +631), IGFBP5 (+1,208 to +1,445), EIF4EBP2 (+564 to +720), MAP3K3 (+841 to +1,022), PNKP (+519 to +728), RAB31 (+315 to +560), RHOQ (+445 to +601), NEUROG1 (+228 to +379), and CDC6 (+1472 to +1981). The RT-PCR products, ranging in size from about 150 to about 510 base pairs, were resolved by electrophoresis in 6% acrylamide gels, cast, and run in 0.25 × TBE (0.0225 mol/l Tris-borate [pH 7.5], 0.5 mmol/l EDTA). Signals were quantified by using a Molecular Dynamics STORM 869 Phosphor Imaging Scanner and Image Quant™ TL software (Amersham Biosciences, Piscataway, NJ, USA).

### Consensus clustering

Several clustering algorithms, including k-means used here, are sensitive to random initialization. To ensure our clustering result was consistent across different initializations and generally robust, we used consensus clustering [102]. We applied consensus clustering to our data by performing 5,000 iterations of k-means clustering, each with a different, random initialization. We used Pearson correlation as the distance metric with k = 8 (see figure of merit [FOM] discussion, below). For every possible pair of genes, we counted the number of cluster results in which that pair was co-clustered. The final step was to derive a consensus clustering result from the average behavior over the entire set of iterations. We applied the average-linkage agglomerative approach, described by Monit and coworkers [102], which uses the fraction of iterations in which each pair of genes co-clustered as a similarity metric and builds a hierarchical linkage tree accordingly. Based on FOM analysis (described below), we picked a similarity threshold that resulted in eight total clusters.

One requirement of the k-means algorithm, which is the basis of our consensus clustering, is that a total number of clusters, k, must be chosen in advance. A common approach to automatically determining k is to use FOM analysis, which attempts to ascertain the minimal number of clusters necessary to produce a good fit of the data to the cluster centroids. Specifically, the FOM for a particular choice of clustering parameters (for instance, the number of clusters) is computed by iteratively withholding a single condition from each expression vector, clustering the data, and measuring how predictive cluster centroids are of all member genes' held-out expression measurement [60]. We used the FOM utility in the MeV Tiger toolbox [103] to perform this analysis and determined that the FOM reached a minimum for k = 8. All figures illustrating clustering results can be interactively viewed on our supplemental website [104] (clustered data can also be downloaded for viewing with Java-Treeview [105]).

### Gene Ontology enrichment analysis

Gene Ontology (GO) analyses were performed in Matlab using a script written by CLM and DLM based on GO TermFinder [106]. Updated gene association and ontology files were downloaded from the GO Consortium database [61] on 24 January 2007, and parsed to create a Matlab reference object for the process ontology. These were used to map Uniprot identifiers to their GO process identifiers. The hypergeometric distribution was used to calculate the significance of GO term assignments in gene clusters. The background set of genes was the set of genes represented on the array, and the *P *values are Bonferroni corrected for multiple hypothesis testing. The Matlab scripts and supporting data can be downloaded from our supplementary website [104]. GO term results were validated and visualized using GOLEM, an interactive tool for browsing the GO [107].

## Additional data files

The following additional data are available with the online version of this paper. Additional file 1 provides dataset shown in Figure 3 (k-means clustered expression data for all genes that pass the fold change and specificity filters). Additional data file 2 shows the validation of microarray data by RT-PCR.

## Supplementary Material

Additional data file 1 Provided is the dataset shown in Figure 3 (k-means clustered expression data for all genes that pass the fold change and specificity filters). Click here for file

Additional data file 2 Shown is the validation of microarray data by RT-PCR. Click here for file

## Figures and Tables

**Table 1:** Cellular functions associated with genes significantly regulated by Ad5 infection

GO term ID	GO term description	P value
Cellular functions associated with down-regulated genes		
GO:0045449	Regulation of transcription	5.97 × e^-06^
GO:0007154	Cell communication	1.63 × e^-04^
GO:0007165	Signal transduction	1.05 × e^-03^
GO:0007275	Development	5.42 × e^-03^
Cellular functions associated with up-regulated genes		
GO:0006281	DNA repair	< 0.00 × e^-11^
GO:0042254	Ribosome biogenesis and assembly	< 0.00 × e^-11^
GO:0007046	Ribosome biogenesis	3.60 × e^-09^
GO:0008380	RNA splicing	4.73 × e^-09^
GO:0006260	DNA replication	1.71 × e^-08^
GO:0007049	Cell cycle	2.03 × e^-08^
GO:0000279	M phase	3.52 × e^-08^
GO:0006913	Nucleocytoplasmic transport	1.12 × e^-05^
GO:0006270	DNA replication initiation	4.37 × e^-05^
GO:0006334	Nucleosome assembly	8.81 × e^-05^
GO:0051169	Nuclear transport	1.86 × e^-04^
GO:0051301	Cell division	1.95 × e^-04^
GO:0031497	Chromatin assembly	7.92 × e^-04^
GO:0009156	Ribonucleoside monophosphate biosynthesis	4.32 × e^-03^
GO:0009124	Nucleoside monophosphate biosynthesis	7.06 × e^-03^

Ad5, adenovirus type 5; GO, Gene Ontology.
