# Supplementary material for: Adenovirus type 5 exerts genome-wide control over cellular programs governing proliferation, quiescence, and survival
Source: Genome Biol. 2007 Apr 12;8(4):R58. doi: 10.1186/gb-2007-8-4-r58 (PMC1896011; doi:10.1186/gb-2007-8-4-r58)
Supplement: Additional data file 2 — Shown is the validation of microarray data by RT-PCR. [file gb-2007-8-4-r58-S2.doc]

| **GENE** | **Log_2_ Relative [RNA]** | | | | | | | | | | | |
| --- | --- | --- | --- | --- | --- | --- | --- | --- | --- | --- | --- | --- |
|  | **Array** | | | | |  | **RT-PCR** | | | | | |
|  | **18** | **24** | **30** | **40** | **54** |  | **18** | **24** | **30** | **40** | **54** | **HRS P.I.** |
| CDC6 | 1.37 | 1.97 | 2.68 | 2.86 | 2.87 |  | 1.62 | 2.71 | 3.04 | 2.41 | 1.50 |  |
| CCNE | 1.74 | 1.98 | 2.23 | 1.66 | 1.21 |  | 1.7 | 1.81 | 1.81 | 1.49 | 0.27 |  |
| EIF4EBP2 | -0.38 | -0.59 | -0.82 | -1.20 | -0.61 |  | 0.06 | -0.02 | -0.03 | -0.43 | -0.71 |  |
| FAM45A | -0.12 | -0.67 | -0.88 | -0.97 | -1.12 |  | 0.08 | -0.04 | -0.03 | -0.24 | -0.44 |  |
| GMNN | 1.73 | 2.31 | 2.66 | 2.08 | 1.57 |  | 1.95 | 2.33 | 2.08 | 1.78 | 1.19 |  |
| IGFBP5 | -0.59 | -0.01 | -0.27 | -1.56 | -1.57 |  | -0.05 | 0.17 | -0.12 | -0.48 | -0.74 |  |
| LENG9 | -0.10 | 0.26 | 0.36 | 1.78 | 3.22 |  | -0.48 | -0.50 | -0.93 | -0.36 | 1.34 |  |
| MAP3K3 | -0.24 | -0.22 | -0.13 | 0.73 | 3.03 |  | -0.20 | -0.01 | 0.26 | 0.10 | 0.38 |  |
| MSH2 | 0.95 | 1.32 | 1.69 | 1.71 | 1.63 |  | 0.92 | 1.59 | 1.42 | 1.39 | 0.67 |  |
| NEUROG1 | 0.19 | -0.23 | 0.15 | 2.70 | 4.12 |  | 0.36 | 0.21 | 0.11 | -0.16 | -0.41 |  |
| NOLC1 | 1.24 | 1.53 | 1.77 | 1.58 | 1.21 |  | 1.08 | 1.22 | 1.09 | 0.95 | -0.32 |  |
| PNKP | -0.07 | 0.06 | 0.16 | 1.21 | 3.97 |  | 0.15 | 0.46 | 0.28 | 0.36 | -0.16 |  |
| RAB31 | -0.21 | -0.87 | -1.00 | -1.12 | -1.20 |  | -0.34 | -0.55 | -0.56 | -0.36 | -1.50 |  |
| SFRP1 | -0.03 | -0.54 | -1.05 | -1.41 | -2.01 |  | 0.21 | 0.06 | -0.10 | -0.54 | -0.75 |  |
